# A novel fast hybrid capture sequencing method for high-efficiency common human coronavirus whole-genome acquisition

**DOI:** 10.1128/msystems.01222-23

**Published:** 2024-04-02

**Authors:** Zixinrong Meng, Shuo Wang, Liping Yu, Kangchen Zhao, Tao Wu, Xiaojuan Zhu, Ning Yang, Qiao Qiao, Junyan Ma, Bin Wu, Yiyue Ge, Lunbiao Cui

**Affiliations:** 1 School of Public Health, Nanjing Medical University, Nanjing, China; 2 NHC Key Laboratory of Enteric Pathogenic Microbiology, Jiangsu Provincial Medical Key Laboratory of Pathogenic Microbiology in Emerging Major Infectious Diseases, Jiangsu Province Engineering Research Center of Health Emergency, Jiangsu Provincial Center for Disease Control and Prevention, Nanjing, China; Argonne National Laboratory, Lemont, Illinois, USA

**Keywords:** coronavirus, hybrid capture, sequencing, MT-capture, multiplex PCR, T-capture

## Abstract

Rapid and accurate sequencing of the entire viral genome, coupled with continuous monitoring of genetic changes, is crucial for understanding the epidemiology of coronaviruses. We designed a novel method called micro target hybrid capture system (MT-Capture) to enable whole-genome sequencing in a timely manner. The novel design of probes used in target binding exhibits a unique and synergistic “hand-in-hand” conjugation effect. The entire hybrid capture process is within 2.5 hours, overcoming the time-consuming and complex operation characteristics of the traditional liquid-phase hybrid capture (T-Capture) system. By designing specific probes for these coronaviruses, MT-Capture effectively enriched isolated strains and 112 clinical samples of coronaviruses with cycle threshold values below 37. Compared to multiplex PCR sequencing, it does not require frequent primer updates and has higher compatibility. MT-Capture is highly sensitive and capable of tracking variants.

**IMPORTANCE:**

MT-Capture is meticulously designed to enable the efficient acquisition of the target genome of the common human coronavirus. Coronavirus is a kind of virus that people are generally susceptible to and is epidemic and infectious, and it is the virus with the longest genome among known RNA viruses. Therefore, common human coronavirus samples are selected to evaluate the accuracy and sensitivity of MT-Capture. This method utilizes innovative probe designs optimized through probe conjugation techniques, greatly shortening the time and simplifying the handwork compared with traditional hybridization capture processes. Our results demonstrate that MT-Capture surpasses multiplex PCR in terms of sensitivity, exhibiting a thousandfold increase. Moreover, MT-Capture excels in the identification of mutation sites. This method not only is used to target the coronaviruses but also may be used to diagnose other diseases, including various infectious diseases, genetic diseases, or tumors.

## INTRODUCTION

Coronaviruses are characterized by non-segmented single-stranded (+) RNA genomes, ranging from 27 to 31 kb, the longest among RNA viruses. Continuous genomic monitoring of the virus *via* whole-genome sequencing enables the recognition of potential adaptive shifts, which facilitates prompt source identification and infection analysis (1). Such scrutiny provides an empirical foundation for assessing the influence of viral mutations on detection and vaccine efficacy and aids in refining prevention and control strategies (1). Targeted sequencing is more cost-effective than metagenomic sequencing (2), due to its focused analysis on specific genomic regions, requiring less sequencing effort and resources (3). Target genome acquisition based on next-generation sequencing (NGS) involves the selective enrichment or capture of specific genomic regions of interest for sequencing (4). The first method is multiplex PCR amplicon-based enrichment (5). This approach relies on PCR amplification with primers designed to specifically target desired genomic regions, an aspect that can reduce its efficiency in detecting emergent mutations (4). The second is traditional liquid-phase hybrid capture (6–8). In this method, specific probes or baits complementary to the target regions are designed and then hybridized to the genomic DNA or RNA (4), thereby capturing the desired sequences; it has the advantages of low design difficulty, high fault tolerance, and high sensitivity. However, this process is very time-consuming, taking 2–3 days from nucleic acid extraction to library capture; at the same time, hybridization capture involves a variety of reagents and a cumbersome operation process (4).

In this study, we design a novel, fast, and high-efficiency method called the micro targets hybrid capture (MT-Capture) system to address the limitations of multiplex PCR sequencing and leverage the advantages of traditional liquid-phase hybrid capture sequencing. The performance of MT-Capture was evaluated using 112 clinical samples of human coronaviruses (HCoVs), particularly clinical samples of SARS-CoV-2. Among the 112 samples mentioned above, 36 samples were sequenced using multiplex PCR and traditional liquid-phase hybrid capture (T-Capture) for comparison. This approach enables researchers to obtain detailed information about the whole viral genome, including all mutations or variants. Such comprehensive data are crucial for understanding viral evolution, recognizing potential adaptive shifts, and aiding in source identification and disease analysis, thereby laying the groundwork for effective disease prevention and control.

## MATERIALS AND METHODS

### Sample source

Positive nasopharyngeal swab clinical samples including SARS-CoV-2, HKU1, 229E, OC43, and NL63, as well as SARS-CoV-2 strains, such as Alpha, Beta, Wuhan, Delta, and Omicron BA.1, were obtained from the Jiangsu Provincial Center for Disease Control and Prevention and sentinel hospitals across various cities in Jiangsu Province. SARS-CoV-2 clinical and strain samples were used to evaluate the sensitivity and accuracy of the MT-Capture system. The Jiangsu Provincial Center for Disease Control and Prevention Ethics Committee approved all study procedures involving human materials.

### Virus extraction and quantification

A total of 200 µL of each sample was processed for nucleic acid extraction (Xi’an Tianlong Biotechnology Co., Ltd, China, #T335) on the automatic nucleic acid extraction instrument GeneRotex 96 (Xi’an Tianlong Biotechnology Co., Ltd, China) in a biosafety level 2 laboratory. Five microliters of each extracted RNA mixed with 7.5-µL nucleic acid amplification reaction solution, 5-µL enzyme mixture, 4-µL primer probes, and 3.5-µL RNase-free water was subjected to reverse transcription polymerase chain reaction (RT-PCR) detection, denaturation (50°C for 10 min, 97°C for 1 min), and 45 amplification cycles (97°C for 5 sec, 58°C for 30 sec) (Jiangsu Bioperfectus Technologies Co., Ltd, China, #JC10223-1N). We performed the following experiments using the cycle threshold (CT) value of the N gene as a reference for viral load. Both the samples and the extracted RNA were stored at −80°C.

### Library preparation for target enrichment

Reverse transcription and library preparation were performed according to the manufacturer’s protocols. The total RNA-to-DNA kit [Nanodigmbio (Nanjing) Biotechnology Co., Ltd, China, #1002402] was employed for the synthesis of complementary DNA (cDNA). Briefly, 5 µL of template RNA fragmentation and random primer binding were carried out at 94°C for 15 min then followed by the cDNA first strand synthesis using the following conditions: 25°C for 10 min, 42°C for 15 min, and 70°C for 15 min. Finally, cDNA second strand synthesis was performed at 16°C for 60 min.

The library was constructed using the DNA Library Preparation Kit (for Illumina) [Nanodigmbio (Nanjing) Biotechnology Co., Ltd, China, #1002103, #1003242]. Initially, 40 µL cDNA was input for end repair and addition of A under the following conditions: 20°C for 30 min and 65°C for 30 min. Then, the adapter ligation process was performed at 20°C for 15 min and 4°C hold. PCR amplification was performed using 20 µL of purified ligation product: 98°C for 2 min, 12 or 14 cycles (98°C for 15 sec, 60°C for 15 sec, and 72°C for 30 sec), 72°C for 2 min, 4°C hold. The number of amplification cycles depends on the CT value of the sample. If CT ≤ 30, 12 cycles of amplification are required. If CT > 30, 14 cycles of amplification are required.

### Micro target hybrid capture

**Fig 1:**
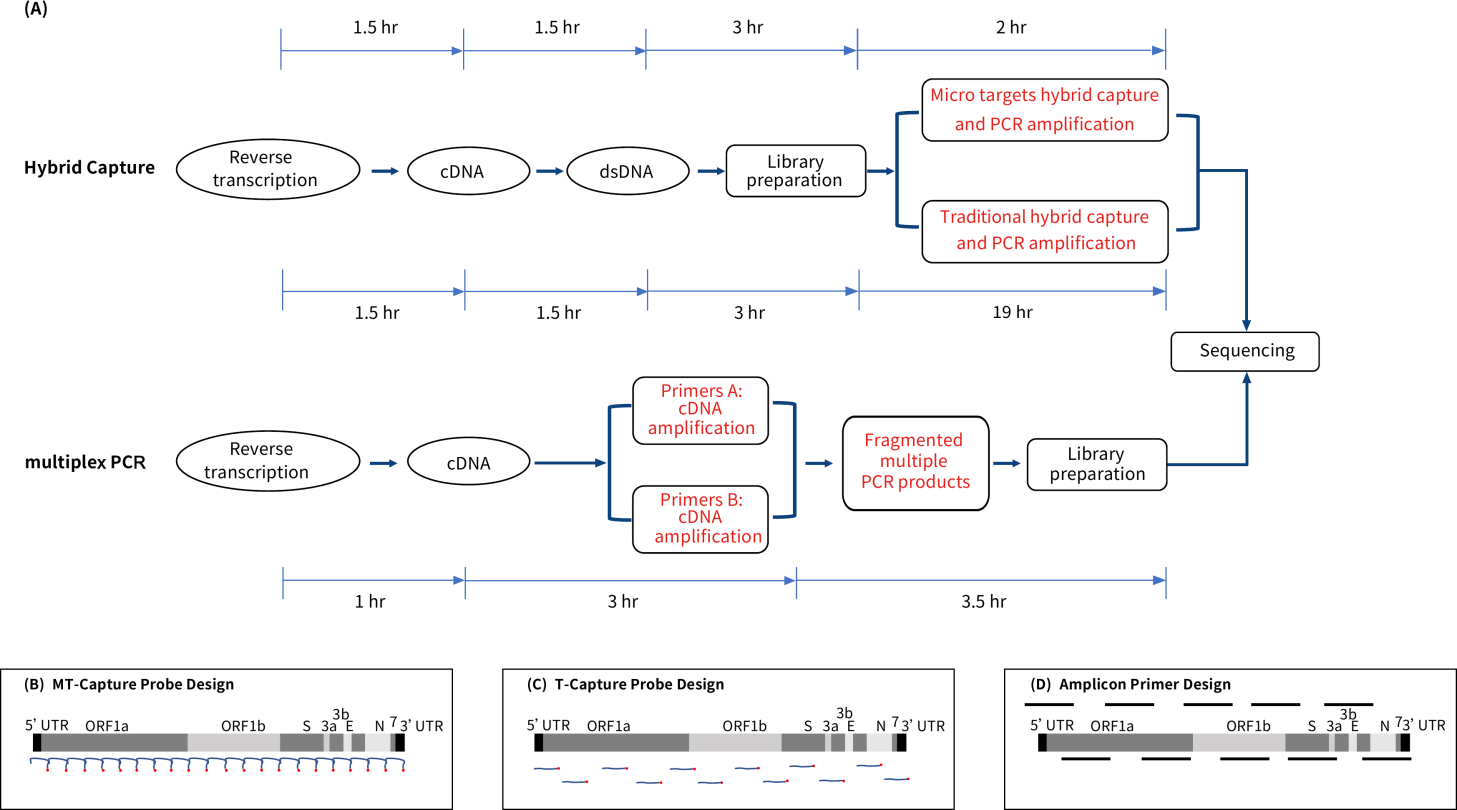
Comparison of three targeted sequencing methods. (**A**) Comparison of the experimental process and the specific time required for whole-genome sequencing using two types of liquid-phase hybrid capture sequencing methods and multiplex PCR sequencing method. (**B**) Design principle of MT-Capture: probes are 20–100-nt non-isolength biotinylated oligonucleotides. Rapid binding of multiple probe conjugation effects for further stabilization. (**C**) Design principle of T-Capture: probes are 120-nt isolength biotinylated oligonucleotides designed by the imbrication principle. (**D**) Design principle of multiplex PCR primers: genome-wide segmental amplification of SARS-CoV-2 nucleic acid using a specific primer pool and high-fidelity enzyme designed by the imbrication principle.

It took 2.5 hours to complete the entire process of MT-Capture (see Fig. 1A), which employed a non-isolength probe set ranging from 20 to 100 nucleotides. “CGTCGGTC” was added to the 5′ end of the probes while “GACCGACG” was added to the 3′ end. So, the 3′ end of one probe was complementary pairing to the 5′ end of another probe, which contributed to composing a “hand-in-hand” conjugation effect between two adjacent probes. Additionally, the 5′ end of the probes is modified with biotin to participate in target binding (see Fig. 1B). The representative genomic sequences of HCoV-HKU1 (NC 006577.2), HCoV-NL63 (NC 005831.2), HCoV-OC43 (LC 687398.1), HCoV-229E (NC 002645.1), and SARS-CoV-2 (NC 045512.2) were used to design probes. The details of all probe sequences are cataloged in Table S1.

The MT-Capture reaction system included the 1 fmol probes, 1× Hyb Buffer: 0.01% bovine serum albumin (BSA) (Sigma), 0.01% Ficoll (Sigma), 0.01% PVP-2 (Sigma), 0.01 M sodium citrate (Sigma) and 0.01 M NaCl (Invitrogen), 1× enhancer: 5× formamide solution (Thermo), 1 µg of human Cot-1 (Thermo), and 100 pmol of blocker (Nanodigmbio). The hybridization reaction was performed by denaturation at 95°C for 2 min and hybridization at 60°C for 1 hour. The reaction system was then supplemented with streptavidin magnetic bead (Thermo) for hybrid capture. Notably, before using magnetic beads, they need to be washed twice with a washing buffer: 80% acetonitrile and 10% RNase-free water; the duration of magnetic streptavidin bead capture was approximately 10 min. Product elution involves the sequential washing of the captured product using three different elution buffers: elution buffer I: 5× SSPE (Sigma) and 1% SDS (Sigma), elution buffer II: 2× SSPE and 0.1% SDS, and elution buffer III: 0.1× SSPE and 0.01% SDS. After the completion of the washing step, amplification was performed with 22.5 µL of resuspended magnetic beads, 25 µL 2× HiFi PCR master mix, and 2.5 µL amplification primer mix II. The reaction conditions were as follows: 98°C for 45 sec, 15 cycles (98°C for 15 sec, 60°C for 30 sec, and 72°C for 30 sec), 72°C for 1 min. Then, amplicons were purified by SP beads (Nanodigmbio), finally eluted with 25 µL TE solution.

### Traditional liquid-phase hybrid capture

The reference sequence for T-Capture probe design was consistent with MT-Capture (NC 045512.2), and T-Capture probes were configured online on the IDT official website (https://sg.idtdna.com/pages). A total of 525 probes were synthesized and supplied by IDT, using a stacked tile method, with each probe comprising 120 nucleotides (see Fig. 1C).

For the T-Capture sequencing, libraries underwent vacuum concentration, followed by liquid-phase hybridization capture facilitated by xGen Hybridization and Wash V2 Kit (Integrated Device Technology, America, #10010354) to assist in completing the whole process. After 16 hours of hybridization, the hybrid products were adsorbed with magnetic streptavidin beads for 45 min and then washed five times to remove any unbound fragments. Finally, PCR amplification was performed while completing the entire process in 25 hours (see Fig. S1; Fig. 1A).

### Library preparation for multiplex PCR sequencing

Reverse transcription and multiplex PCR amplification were performed following the manufacturer’s protocols [Baiyi Two-Step Method Long Fragment SARS-CoV-2 Whole-Genome Capture (Baiyi Technology Co., Ltd, China, #BK-WCoV024IITS) and Illumina COVIDSeq Assay (Illumina, Inc, America, #20049393)], in which the primers were updated in time, suitable for all SARS-CoV-2 subtypes. Subsequently, library preparation was executed for Baiyi’s PCR products utilizing ILMN DNA LP(M) Tagmentation (Illumina, #20060059) and concurrently for Illumina’s PCR products *via* the COVIDSeq Assay. The entirety of this process demanded approximately 7.5 hours (see Fig. 1A).

### Sequencing

The samples were sequenced using the Illumina MiniSeq High Output Reagent Cartridge (300 cycles) (Illumina, #15073286) and Illumina MiniSeq Flow Cell (Illumina, #15073184) on the Illumina MiniSeq sequencing platform.

### Bioinformatics analysis

Firstly, sequencing read pools were combined (as R1 and R2), Illumina adapter sequences were removed, and low-quality sequences were trimmed or removed using fastp (0.21.0) (9). The genome of coronavirus was assembled, and the sequencing depth was calculated using the Iterative Refinement Meta-Assembler software (v1.0.3) (10). Subsequently, coverage was calculated by comparing it to the reference genome. In analyzing multiplex PCR sequencing FASTQ data, fastp 0.21.0 was used to eliminate 30 nucleotides from each side; this step was not essential for liquid-phase hybrid capture. Alignment with the reference genome was conducted using the BWA (0.7.17-r1188) (11) software, followed by mutation calling *via* bcftools (htslib 1.11) (12). The snpEff (5.0e) (13) tool was employed for mutation annotation. Finally, data visualization was executed using the ggplot2 (3.4.1) (14) package in R package. The default parameters were used for analysis.

## RESULTS

### Comparison of design principles and workflow for three sequencing preprocessing methods

Multiplex PCR sequencing is a method that involves designing primers for specific target regions in the genome (see Fig. 1D). However, as new mutations emerge, reassessing and redesigning the primer sequences may be necessary (15).

Liquid-phase hybrid capture methods encompass the affinity between biotinylated probes and the sequence of interest. The high tolerance for mismatches enables the successful capture of highly diverse sequences (16), thus facilitating the discovery of diverse mutation types. However, the T-Capture process is complex and lacks stability. In contrast, the probe of the MT-Capture system mainly consists of three parts. The middle section is the target binding region, while the 5′ and 3′ sections are conjugated regions, which means the 5′ end of one probe can complementarily pair with the 3′ end of another probe and the 3′ end can complementarily pair with the 5′ end of another probe. The length of the binding region between the probe and the target is longer than that of the conjugated region. Thus, the conjugation effect occurs after the binding of probes and targets. This effect lasts throughout the entire process of hybrid capture, mainly stabilizing the binding between probes and targets. Each probe is labeled with biotin groups at its 5′ end, and since the average length of MT-Capture probes is shorter than that of T-Capture probes, MT-Capture has a larger quantity of biotin groups. The conjugation effect as well as more biotin groups accelerates the speed and efficiency of the binding of streptavidin-coated magnetic beads to the targets. This innovation simplifies both the hybridization and elution processes, consequently reducing the experimental time to levels analogous to multiplex PCR sequencing (see Fig. 1A; Table 1).

**TABLE 1:** Comparison of three different sequencing methods

Item	Hybrid capture		Multiplex PCR
	MT-Capture	T-Capture	
Probe design scheme	20–100-nt non-isolength biotinylated oligonucleotides	120-nt biotinylated oligonucleotides	200–300-nt or 1,200-nt amplicon
Duration of the entire process	8 hours	25 hours	7.5 hours
Operational convenience	Simple	Complicated	Simple
Detection sensitivity	High	High	Low
Tracking of subtypes	Y	Y	N
Sample multiplexing	Y	Y	N

### MT-Capture sequencing can track diverse SARS-CoV-2 subtypes

**Fig 2:**
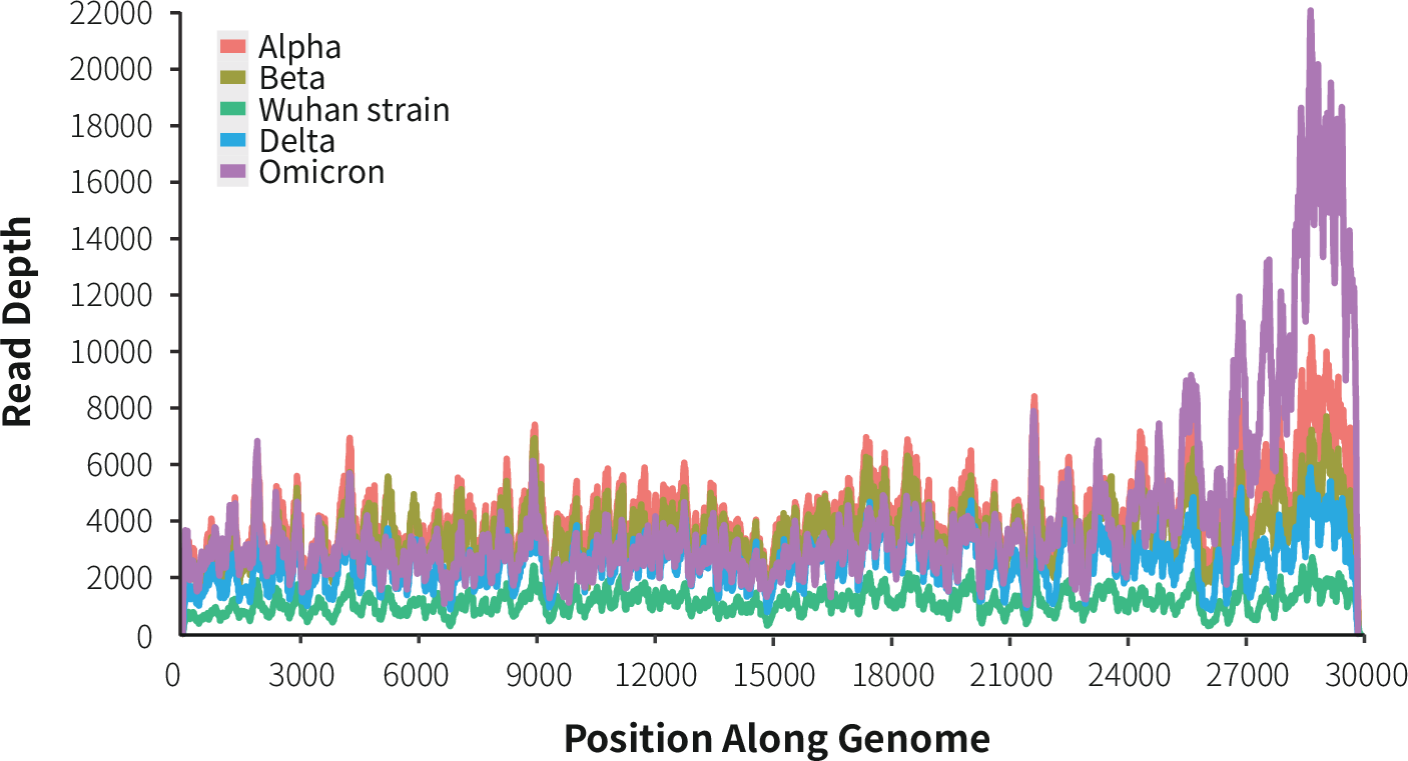
MT-Capture can track different variants; this figure shows the sequencing depths and whole-genome coverage of five different strains of SARS-CoV-2 including Alpha, Beta, Wuhan, Delta, and Omicron BA.1.

MT-Capture sequencing, initially derived from the original Wuhan strain, can effectively track the evolving variations in SARS-CoV-2. This is made possible by the fault tolerance of hybrid capture probes. In addition to the Wuhan strain, different variants such as Alpha, Beta, Delta, and Omicron BA.1 have been used as templates, each providing complete genome coverage (see Fig. 2). The hybrid capture probes offer flexibility in monitoring diverse mutation patterns within the pathogen.

### MT-Capture sequencing reveals higher detection sensitivity

**Fig 3:**
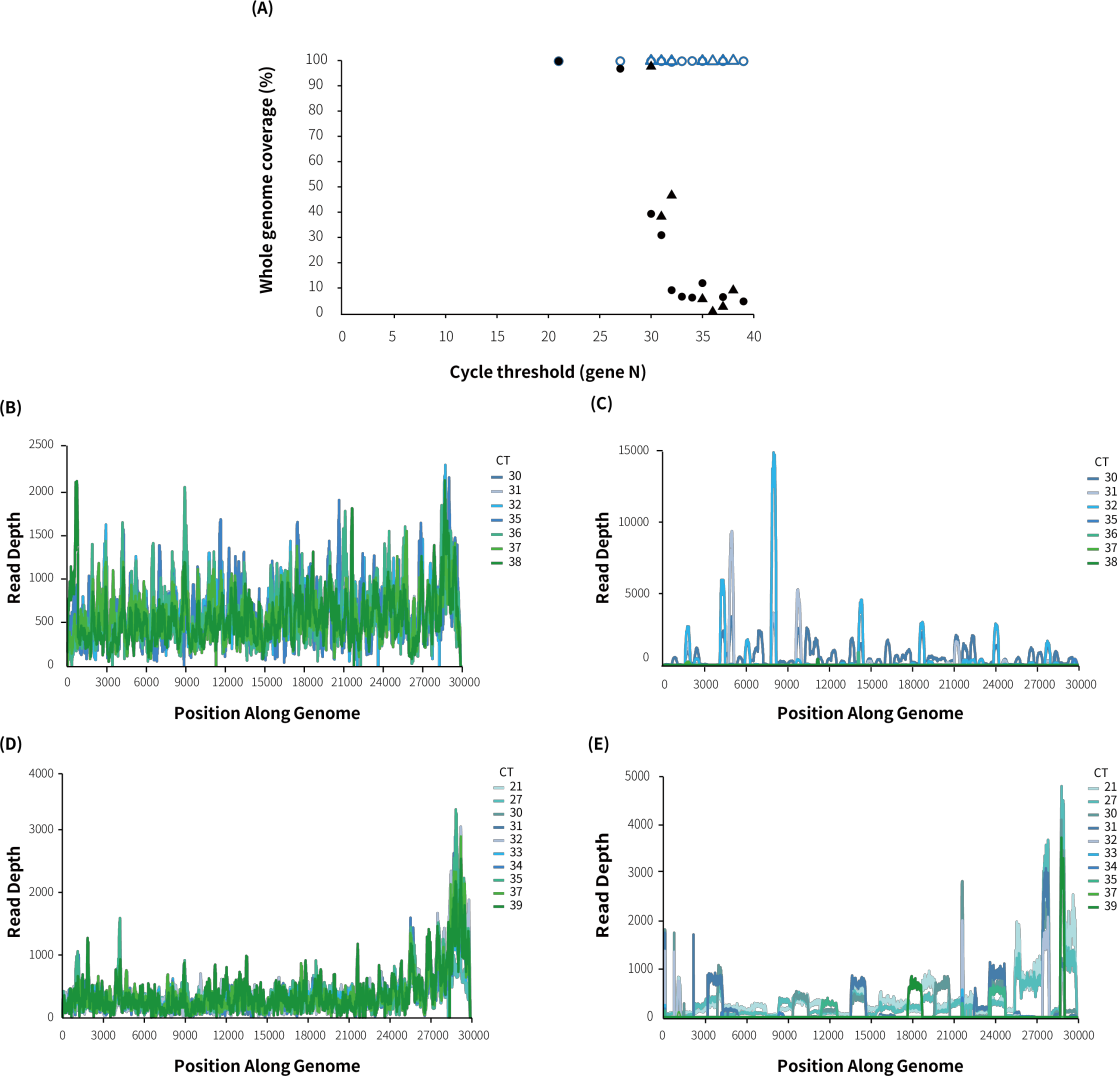
Comparison of detection sensitivity between MT-Capture sequencing and multiplex PCR sequencing. (**A**) Using the CT value of N gene obtained by RT-PCR as an indicator of viral load of virus serial dilution samples, the whole-genome coverage of MT-Capture and multiplex PCR under different CT values was compared. △, Delta strains enriched by MT-Capture; ○, Omicron strains enriched by MT-Capture; ▲, Delta strains amplified by multiplex PCR; ●, Omicron strains amplified by multiplex PCR. (**B**) and (**C**) Coverage and sequencing depth of Delta samples with different CT values for MT-Capture sequencing and multiplex PCR sequencing, respectively; (**D**) and (**E**) coverage and sequencing depth of Omicron BA.1 samples with different CT values for MT-Capture sequencing and multiplex PCR sequencing, respectively.

For comparing the efficacy of multiplex PCR sequencing and MT-Capture sequencing with different SARS-CoV-2 viral loads, gradient-diluted Omicron BA.1 and Delta samples (main viruses that caused an epidemic in China except for the Wuhan strain) were utilized. MT-Capture demonstrates high sensitivity and is capable of assembling whole-genome sequences even at extremely low viral loads (see Fig. 3A). In contrast, multiplex PCR sequencing struggles to amplify whole-genome sequences from samples with high CT values. However, MT-Capture is able to sequence the whole genome for samples with CT values between 32 and 38. Despite the low viral load, MT-Capture can achieve whole-genome coverage and uniform sequencing depth compared to multiplex PCR (see Fig. 3B through E).

### MT-Capture is superior to multiplex PCR sequencing and T-Capture sequencing in the accuracy and sensitivity of detecting mutation sites of SARS-CoV-2

The effectiveness of three different sequencing methods, namely, MT-Capture, T-Capture, and multiplex PCR, was examined using 36 clinical samples. The study focused on on-target rates, genome coverage, and mutation detection (see Table S2). Particularly, MT-Capture exhibited a superior capacity in identifying mutation sites in *ORF1ab*, *S*, *ORF3a*, *M*, *ORF7a*, *ORF7b*, and *N* genes compared to T-Capture and multiplex PCR (see Table 2; Table S3).

**TABLE 2:** Summary of the number of mutation sites

Gene^*a*^	Missense mutation^*b*^			Synonymous mutation^*c*^			Common mutation^*d*^		
	MT-Capture	Multiplex PCR	T-Capture	MT-Capture	Multiplex PCR	T-Capture	MT-Capture & multiplex PCR	MT-Capture & T-Capture	T-Capture & multiplex PCR
5′UTR	0	0	0	0	0	0	54	71	54
ORF1ab	477	405	470	304	248	293	677	785	671
S	926	821	897	25	25	24	785	842	769
ORF3a	33	28	32	38	38	38	66	70	65
E	24	24	24	0	0	0	24	24	24
M	81	74	79	19	2	19	75	97	75
ORF7b	12	8	13	23	23	22	31	34	30
ORF6	2	2	2	5	5	5	7	7	7
ORF7a	54	50	51	5	5	5	55	56	55
ORF8	2	2	2	0	0	0	15	15	15
N	214	173	211	45	45	45	211	222	215
ORF10	0	0	0	15	14	15	14	15	14
3′UTR	0	0	0	0	0	0	32	48	33

^*a*^ Gene, different genome names of SARS-CoV-2.

^*b*^ Missense mutation, a common single-nucleotide variation (SNV) that describes a change in a single nucleotide in the genome, resulting in a change in an amino acid in the corresponding protein sequence.

^*c*^ Synonymous mutation, a SNV that refers to a change in a single nucleotide in the genome, but due to codon redundancy, this change does not result in a change in the amino acid sequence of the gene encoding the protein. Therefore, this mutation will not directly affect the structure and function of the protein.

^*d*^ Same variant, the number of mutation sites with consistent detection results in both methods. Display sites with higher than 50% mutation frequency and sequencing depth >50.

**Fig 4:**
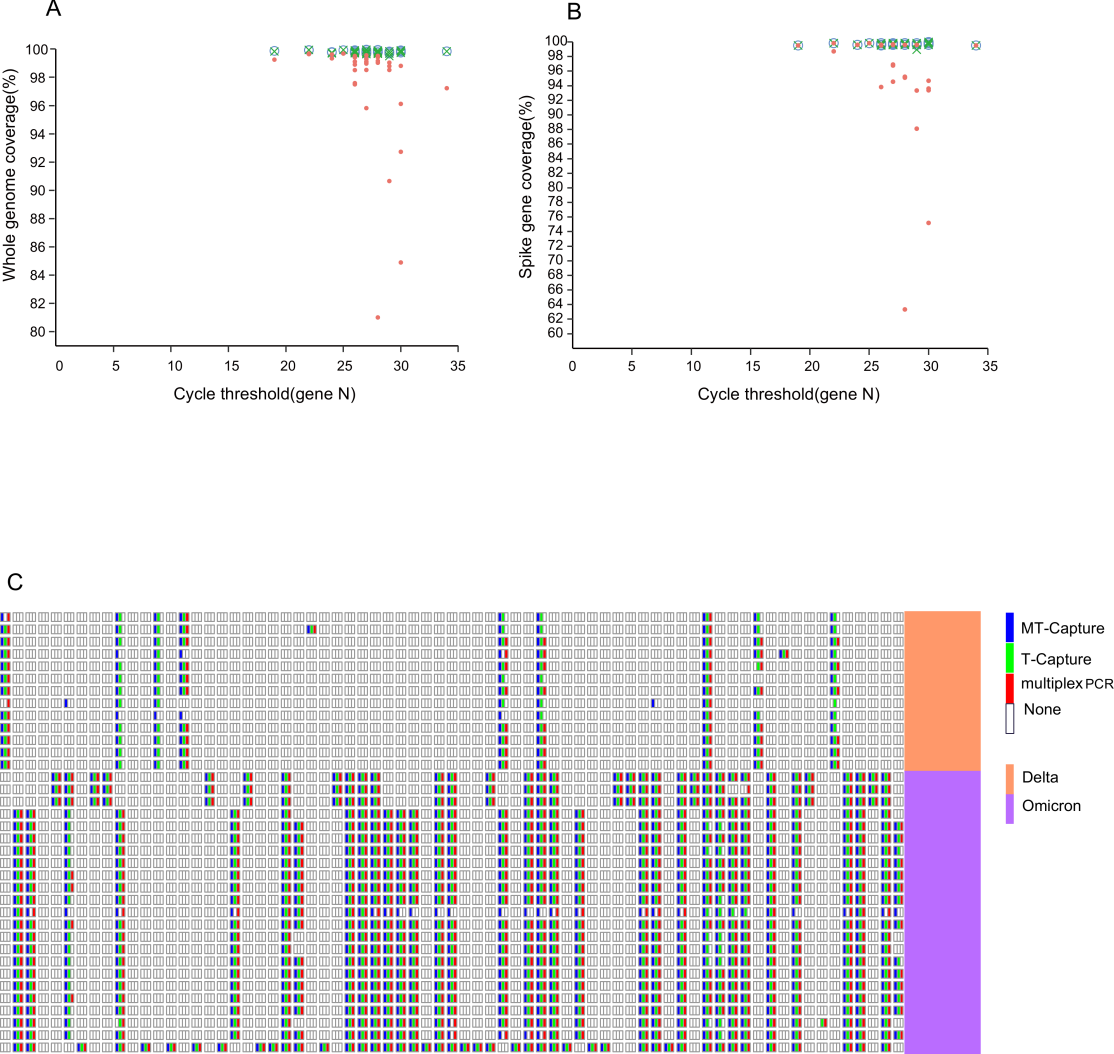
Comparison of three methods in coverage and mutation site detection. Using the CT value of the N gene obtained by RT-PCR as an indicator of viral load of clinical samples, compare (**A**) 36 clinical samples’ whole-genome coverage and (**B**) 36 clinical samples’ spike gene coverage of MT-Capture (white circle), T-Capture (green x), and multiplex PCR (red circle) under different CT values. (**C**) S1 gene mutation analysis in clinical samples sequenced by MT-Capture, T-Capture, and multiplex PCR. The detection sites are arranged in order of S gene 5′−3′ (see supplementary material for site information). Downsample all data to the same amount of data. Display sites with mutation frequency higher than 50% and sequencing depth >50.

We compared genome coverage by the three sequencing methods at different viral loads with 36 clinical samples. The MT-Capture sequencing and T-Capture sequencing exhibit higher coverage of the whole genome than the multiplex PCR sequencing (see Fig. 4). Specifically, for samples with CT values ≥29, whole-genome coverage for MT-Capture, T-Capture, and multiplex PCR was 99.80%, 99.77%, and 95.20%, respectively. The gradient dilution results, combined with average on-target rates of 70.23%, 53.40%, and 58.05% for the respective methods, highlight that evaluating sequencing methods based solely on target rates is not comprehensive. Therefore, an integrated analysis with genome coverage is required. The utilization of 0.2× mean for assessing whole-genome coverage uniformity revealed 99.76%, 99.49%, and 61.91% uniformity for MT-Capture, T-Capture, and multiplex PCR methods, respectively. With higher coverage uniformity, MT-Capture performed better than the other methods, especially when compared to multiplex PCR sequencing. Additionally, a detailed comparison of the sequencing depth of five distinct samples (210806-48, 210806-51, 221219-31, 230303-52, and 221227-44) substantiated the enhanced coverage uniformity of MT-Capture and T-Capture over multiplex PCR sequencing (Fig. S2).

Sequencing results indicated 477 and 304 missense and synonymous mutations, respectively, in ORF1ab by MT-Capture, compared to 470 and 293 by T-Capture and 405 and 248 by multiplex PCR. While MT-Capture detected 214 missense mutations within the N gene, T-Capture and multiplex PCR detected 211 and 173, respectively, with synonymous mutations consistent across all three methods.

The *S* gene (21563-25384) contains the most informative segment for determining related SARS-CoV-2 variants (17). New mutations primarily accumulate in the *S* protein, which is the basis for many vaccine developments (18, 19). In these 36 clinical samples, MT-Capture detected 926 missense mutations and 25 synonymous mutations, T-Capture detected 897 missense mutations and 24 synonymous mutations, and multiplex PCR detected 821 missense mutations and 25 synonymous mutations. A detailed analysis was performed on the spike S1 mutations across 36 clinical samples employing the three sequencing methods (Fig. 4). For samples with CT values greater than 25, both MT-Capture and T-Capture provided better coverage of the S gene than the multiplex PCR method. MT-Capture hybrid capture can detect mutation sites more than T-Capture and multiplex PCR. Partial samples were validated using first-generation Sanger sequencing. For example, sample 210806-50 showed a G base at position 21757 when detected using multiplex PCR sequencing. In contrast, Sanger sequencing and liquid-phase hybrid capture sequencing detected a C base at the same position. Similarly, while multiplex PCR sequencing detected a G base at position 21987 in 10 samples, both Sanger sequencing and liquid-phase hybrid capture sequencing identified it as an A base. Furthermore, some regions revealed mutations by MT-Capture but were not amplified by multiplex PCR, leading to undetected mutations (Fig. S3).

### Utilization of MT-Capture sequencing for clinical sample analysis

Using the MT-Capture hybrid capture method, we sequenced 112 clinical samples (including 36 samples used for the above comparison of three sequencing methods) (Table S4). The MT-Capture method achieved whole-genome coverage in all sequenced samples of 229E, OC43, NL63, HKU1, and SARS-CoV-2 (Fig. S4). Despite low viral loads, with CT values of 32, 34, and 37, MT-Capture performed well (Fig. S5). A phylogenetic tree constructed from the S gene of the SARS-CoV-2 samples (Fig. S6) illustrated the genetic variations and the diversity within the SARS-CoV-2 viral genome, confirming the utility of this hybridization capture method. This method enables detecting and monitoring different types of coronaviruses and their strains within the same reaction system.

## DISCUSSION

Coronaviruses can cause severe respiratory diseases in humans. Since the emergence of SARS-CoV-2 in 2019, numerous strains and variants of this virus have been identified, garnering significant attention in the scientific community (20–24). Continuous scrutiny of the genomic characteristics of the pathogen is essential for managing infectious diseases. Targeted sequencing can provide a cost-effective method for data generation for many comparative genomic applications; such genomic data facilitate various scientific investigations, including identifying pathogens, analyzing key genes, tracking origins, conducting dynamic surveillance, and making epidemiological projections.

The multiplex PCR sequencing method, widely accepted in global laboratories, functions efficiently with samples with typical viral loads (CT ≤ 32). However, it has several limitations. For instance, it often results in the loss of regions near the 5′ or 3′ ends and might miss novel viral mutations outside the designated degenerate base range. These may be attributed to the method’s reliance on specific primer sequences, leading to mismatches during amplification, substantial gaps in genome assembly, and impediments to classifying genomic clusters or identifying noteworthy variants of concern. Moreover, lower viral load samples present uneven amplification and coverage irregularities (25, 26), thus posing difficulties in sequencing, especially in samples with elevated CT values (27–30). Although traditional liquid-phase hybrid capture can achieve uniform and comprehensive genome coverage in targeted regions of the genome with low viral load (31), the entire experimental process is labor-intensive and time-consuming.

In routine practice, non-target elements might be captured during the hybridization phase; hence, a higher on-target rate equates to improved data efficiency in liquid-phase hybrid capture. Viral load also influences this variable, with lower CT values leading to an increased on-target rate. The MT-Capture consistently outperformed the T-Capture in our results. Nevertheless, while multiplex PCR displays superiority over liquid-phase hybrid capture in the on-target rate, it necessitates a balanced consideration of both on-target rate and uniformity of coverage. Although there can be uneven coverage in different viral genome regions, this does not necessarily compromise the overall target efficiency. In some cases, liquid-phase hybridization may produce a lower on-target rate than multiplex PCR yet offer superior whole-genome coverage, potentially due to multiplex PCR’s heterogeneous coverage.

In addition, this study demonstrated that MT-Capture outperforms multiplex PCR sequencing in SARS-CoV-2 evolution monitoring, as it can capture the whole-genome sequence of different subtypes of SARS-CoV-2. The higher fault tolerance of liquid-phase hybrid capture sequencing results from different design principles, allowing for more robust identification of variants. In the context of SNV identification, MT-Capture sequencing was superior to T-Capture sequencing. It identified more mutations and showed greater precision in mutation site detection, a finding supported by Sanger sequencing.

In conclusion, the cutting-edge MT-Capture probe method is instrumental in SARS-CoV-2 enrichment and variant research. The continuous innovations in genome sequencing are revolutionizing epidemiology, enhancing efficiency and accuracy, and fortifying the response and readiness for disease outbreaks (32). The emergence of novel methods like MT-Capture enables precision in epidemiological interventions and public health strategies, extending beyond human coronavirus to other microbial pathogens, human cancers, and even complex environmental samples. Consequently, this method’s heightened sensitivity can be harnessed to augment monitoring efforts, thus potentially averting or alleviating future epidemics.

## Data Availability

The raw sequence data reported in this paper have been deposited in the Genome Sequence Archive (Genomics, Proteomics & Bioinformatics 2021) in National Genomics Data Center (Nucleic Acids Res 2022), China National Center for Bioinformation / Beijing Institute of Genomics, Chinese Academy of Sciences (GSA: CRA011938) that are publicly accessible at https://ngdc.cncb.ac.cn/gsa.

## SUPPLEMENTAL MATERIAL

FIG. S1 msystems.01222-23-s0001.tif The specific hybridization processes of MT-Capture and T-Capture.

FIG. S2 msystems.01222-23-s0002.tif Compare the sequencing depth of five samples.

FIG. S3 msystems.01222-23-s0003.tif Comparison of S1 gene sequencing results between three methods and Sanger sequencing results.

FIG. S4 msystems.01222-23-s0004.tif Genome coverage of four different human coronaviruses.

FIG. S5 msystems.01222-23-s0005.tif The whole genome coverage of MT-Capture sequencing in all clinical samples with different CT values.

FIG. S6 msystems.01222-23-s0006.tif Maximum likelihood tree based on the S gene for all clinical samples sequenced using MT-Capture sequencing.

Legends msystems.01222-23-s0007.docx Supplemental figure legends.

Supplemental Tables msystems.01222-23-s0008.xlsx Tables S1 and S2.

Table S3 msystems.01222-23-s0009.xlsx Specific mutation sites discovered in clinical samples using three different methods.

Table S4 msystems.01222-23-s0010.xlsx All clinical samples information sequenced using MT-Capture sequencing.

ASM does not own the copyrights to Supplemental Material that may be linked to, or accessed through, an article. The authors have granted ASM a non-exclusive, world-wide license to publish the Supplemental Material files. Please contact the corresponding author directly for reuse.
