# Supplementary figures and images for: A novel fast hybrid capture sequencing method for high-efficiency common human coronavirus whole-genome acquisition
Source: mSystems. 2024 Apr 2;9(5):e01222-23. doi: 10.1128/msystems.01222-23 (PMC11097644; doi:10.1128/msystems.01222-23)

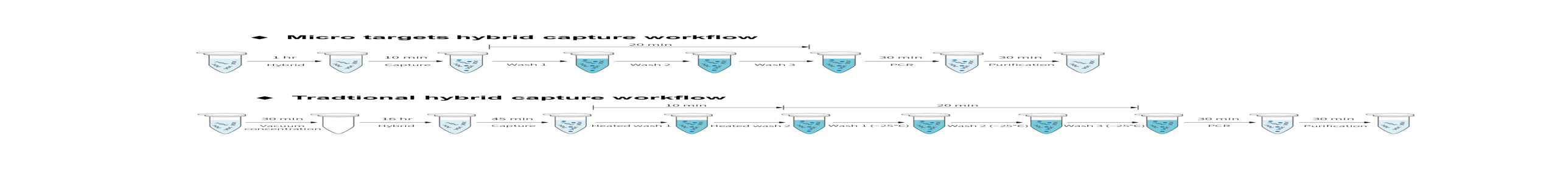

Supplement: FIG. S1 — The specific hybridization processes of MT-Capture and T-Capture. [file msystems.01222-23-s0001.tif]

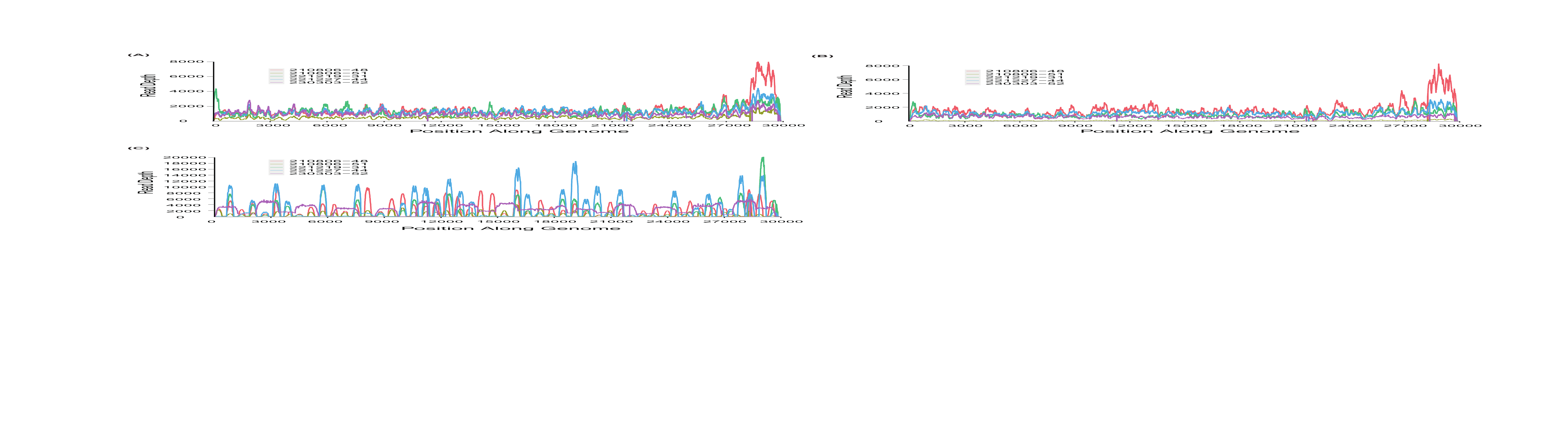

Supplement: FIG. S2 — Compare the sequencing depth of five samples. [file msystems.01222-23-s0002.tif]

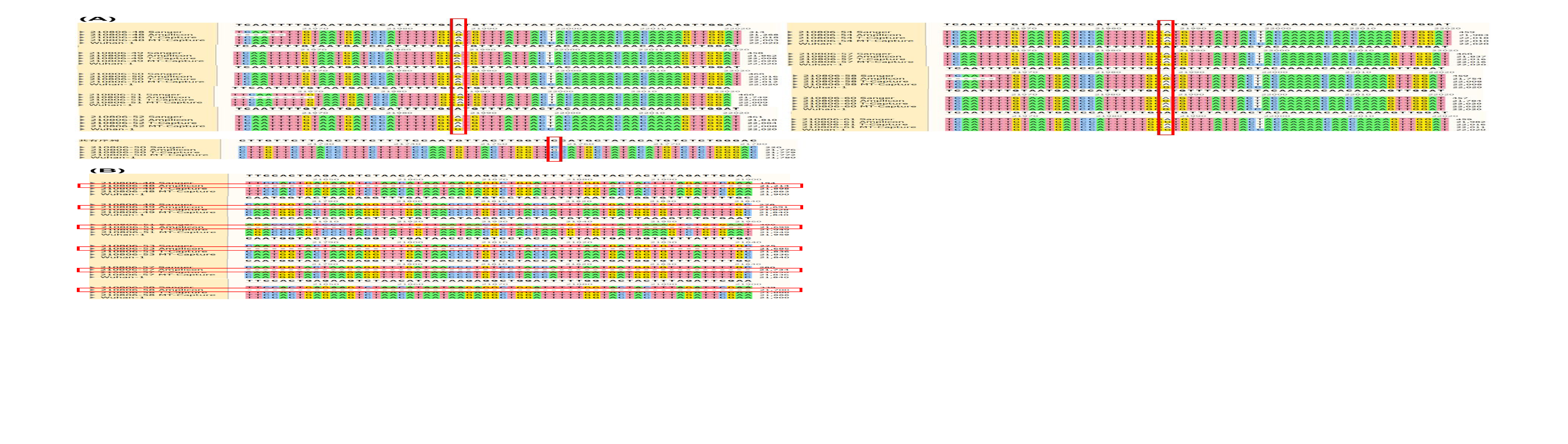

Supplement: FIG. S3 — Comparison of S1 gene sequencing results between three methods and Sanger sequencing results. [file msystems.01222-23-s0003.tif]

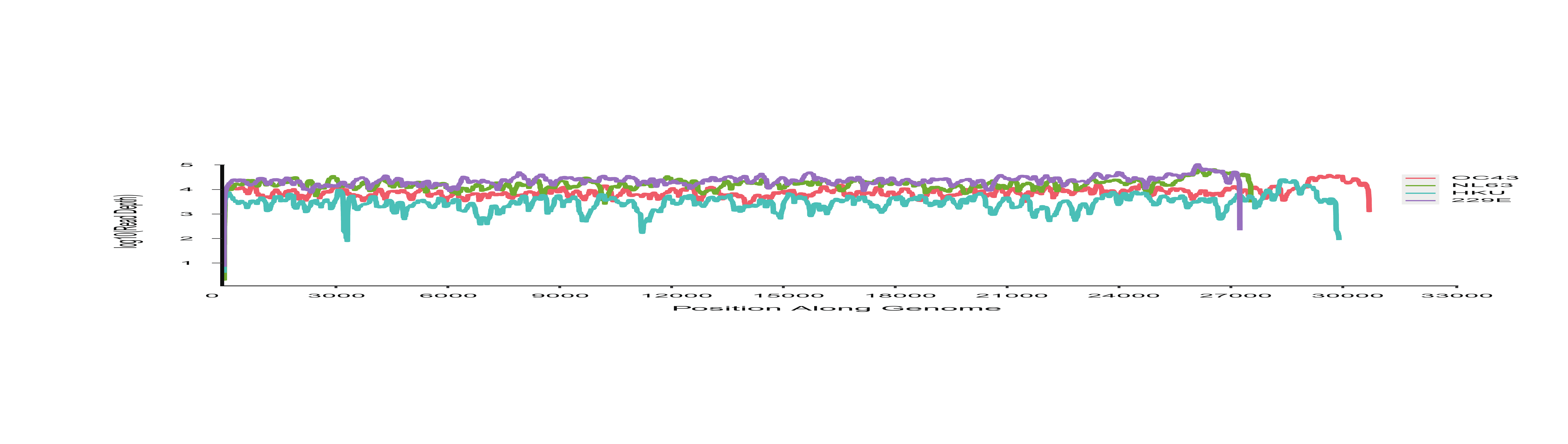

Supplement: FIG. S4 — Genome coverage of four different human coronaviruses. [file msystems.01222-23-s0004.tif]

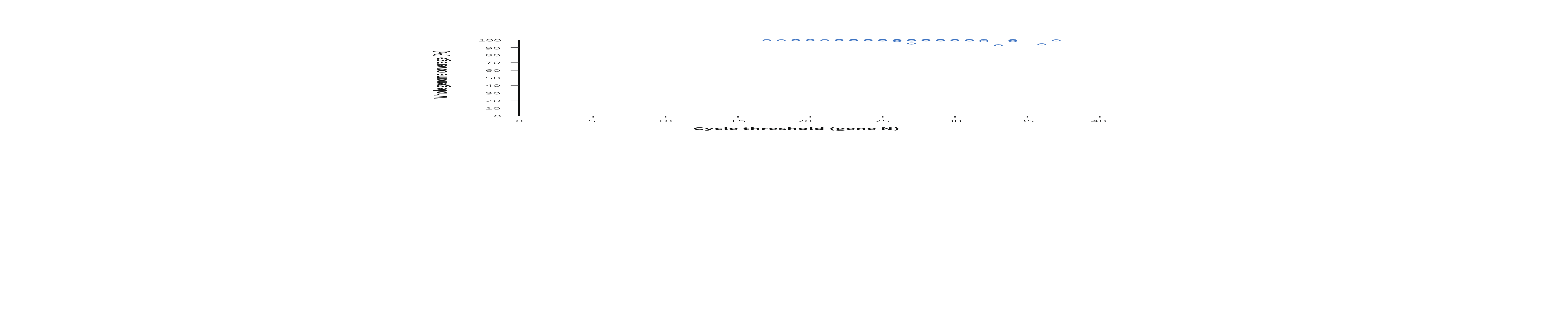

Supplement: FIG. S5 — The whole genome coverage of MT-Capture sequencing in all clinical samples with different CT values. [file msystems.01222-23-s0005.tif]

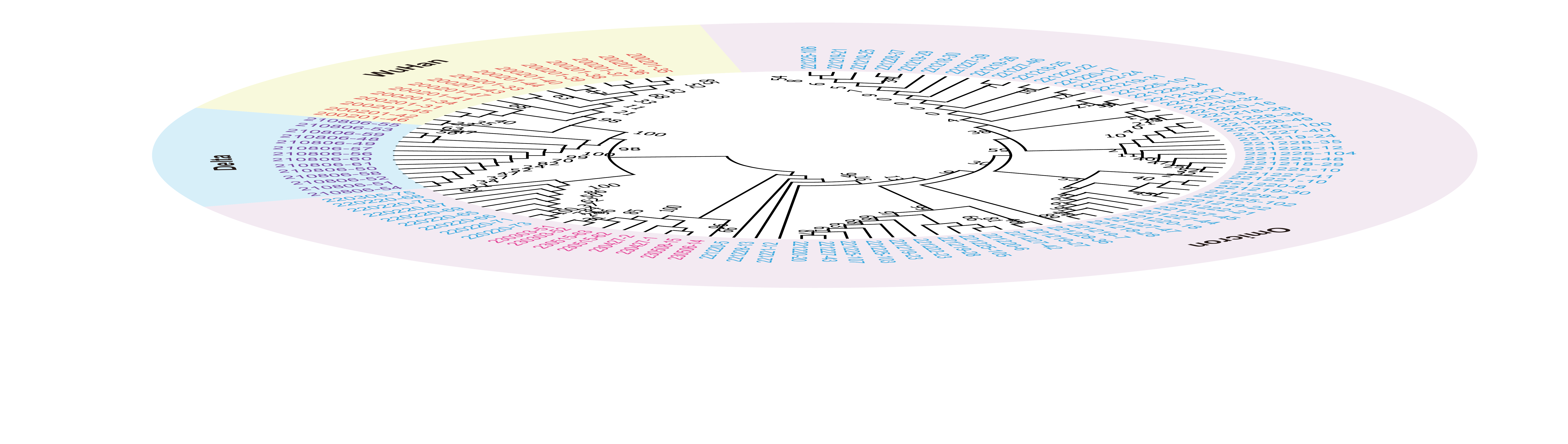

Supplement: FIG. S6 — Maximum likelihood tree based on the S gene for all clinical samples sequenced using MT-Capture sequencing. [file msystems.01222-23-s0006.tif]
