# Supplementary material for: A novel fast hybrid capture sequencing method for high-efficiency common human coronavirus whole-genome acquisition
Source: mSystems. 2024 Apr 2;9(5):e01222-23. doi: 10.1128/msystems.01222-23 (PMC11097644; doi:10.1128/msystems.01222-23)
Supplement: Legends — Supplemental figure legends. [file msystems.01222-23-s0007.docx]

**Supplementary Figure Legends**

**Supplementary FIG 1.** The specific hybridization processes of MT-Capture and T-Capture.

**Supplementary FIG 2.** Compare the sequencing depth of five samples (210806-48, 210806-51, 221219-31, 230303-52, and 221227-44): (A) Clinical samples sequencing depth using MT-Capture sequencing (B) Clinical samples sequencing depth using T-Capture sequencing (C) Clinical samples sequencing depth using multiplex PCR sequencing.

**Supplementary FIG 3.** Comparison of S1 gene sequencing results between three methods and Sanger sequencing results. (A) Base substitution (B) Some sequences have not been amplified by multiplex PCR.

**Supplementary FIG 4.** Genome coverage of four different human coronaviruses.

**Supplementary FIG 5. The whole genome coverage of MT-Capture sequencing in all clinical samples with different CT values.** The CT values obtained by RT-PCR were used as indicators of 108 clinical samples’ viral load, and hundreds of samples were used to demonstrate the performance of MT-Capture.

**Supplementary FIG 6.** Maximum likelihood tree based on the S gene for all clinical samples sequenced using MT-Capture sequencing.
